# Monitoring of patients with rheumatoid arthritis by indocyanine green (ICG)-enhanced fluorescence optical imaging treated with anti-TNFα therapy

**DOI:** 10.1186/s13075-022-02795-w

**Published:** 2022-05-21

**Authors:** S. Hertrampf, J. Klotsche, Q. Schefer, A. M. Glimm, G. R. Burmester, P. Hoff, G. Schmittat, T. Häupl, S. Hermann, M. Backhaus, Sarah Ohrndorf

**Affiliations:** 1 Department of Rheumatology and Clinical Immunology, Charité – Universitätsmedizin Berlin, 10117 Berlin, Germany; 2 Deutsches Rheumaforschungszentrum (DRFZ) Berlin, Leibniz Research Network, Charité – Universitätsmedizin Berlin, Berlin, Germany; 3 Institute for Social Medicine, Epidemiology and Health Economics, Charité – Universitätsmedizin Berlin, Berlin, Germany; 4 regenold GmbH, Zöllinplatz 4, Badenweiler, Germany; 5 Endokrinologikum Berlin am Gendarmenmarkt, Berlin, Germany; 6 Department of Internal Medicine - Rheumatology and Clinical Immunology, Park-Klinik Weißensee, Berlin, Germany

**Keywords:** Fluorescence optical imaging, Anti-TNFα therapy, Certolizumab, Rheumatoid arthritis, Inflammation

## Abstract

**Background:**

Fluorescence optical imaging (FOI) enables visualisation of inflammation in both hands in rheumatoid arthritis (RA).

**Objective:**

To investigate the usefulness of FOI in treatment monitoring under anti-TNFα therapy with certolizumab pegol (CZP) in patients with RA in comparison to clinical and laboratory outcome parameters.

**Methods:**

CZP-naïve patients with RA were eligible for this open-label study with an observational period of 52 weeks. Disease activity was monitored by the clinical score DAS28, tender/swollen joint count (TJC-28/SJC-28) and laboratory outcomes for systemic inflammation (CRP and ESR). FOI results were analysed in three different phases (P1-3) and PrimaVistaMode (PVM) by the FOI activity score (FOIAS).

**Results:**

Twenty-eight RA patients (median age 52.5 years, 26 females, thirteen with a history of other biologic therapy) were included. DAS28 (CRP) decreased from moderate disease activity at baseline (median 4.6, IQR 1.8) to low disease activity at week (w)52 (median 2.7, IQR 2.1; *p* < 0.001). Statistically significant decreases could also be demonstrated for SJC-28 and TJC-28. CRP/ESR were reduced numerically from baseline to w52. FOIAS in P1 (early phase) showed a continuous decrease of enhancement during the course of treatment period: from baseline (median 1.5, IQR 9.3) over w6 (median 1.0, IQR 3.0; *p* = 0.069), w12 (median 0.5, IQR 3.0; *p* = 0.171), w24 (*n* = 27, median 0.0, IQR 3.0; *p* = 0.004), until w52 (*n* = 18, median 0.0, IQR 2.8; *p* = 0.091), which could not be presented for FOIAS in P2, P3 and PVM.

**Conclusion:**

FOI in P1 appears to be a valuable tool for fast and easy monitoring of treatment response to certolizumab in a clinical setting.

## Introduction

Rheumatic and musculoskeletal diseases (RMDs) represent a heterogeneous group with a high prevalence of around 22% in adults in the United States (US) [1]. Even though there is a wide range of novel therapeutics, inflammatory arthritis still causes reduced mobility, limited quality of life and high health care costs [1, 2]. Among RMDs, rheumatoid arthritis (RA) is the most common, with a prevalence of 0.5–1.0% [3, 4]. To reduce the effect of chronic joint inflammation, present therapeutic strategies focus on early recognition during the “window of opportunity“ for effective therapy to improve short and long term outcomes significantly [5, 6].

Inhibitors of tumour necrosis factor alpha (TNFα) were the first developed among biological medications and they dramatically changed the therapeutic perspectives of RA patients [7]. Follow-up studies investigating their clinical and radiographic effects have shown that TNFα inhibitors, especially in combination with conventional synthetical disease-modifying antirheumatic drugs (csDMARD), lead to significant improvements in clinical status and significant inhibition of radiographic progression.

In order to monitor the therapeutic response in RA, several options are applied, such as different clinical disease activity scores, e.g. DAS28 [8]. For early and more objective monitoring of RA disease activity, musculoskeletal ultrasound (MSUS) and magnetic resonance imaging (MRI) are used in clinical practice [9–12]. In several clinical studies, MSUS presented sensitivity in disease changes under therapy, especially in power Doppler mode (PD)-detected synovitis and tenosynovitis [13, 14]. Different joint combinations have been used for MSUS scoring. The US7-joint score first proposed by Backhaus et al. has been the most utilised score in the literature and includes five hand and two foot joints of the clinically dominant side [15].

More recently, after its first report in 2006 [13] and the launch of a commercial device in 2009 (Xiralite GmbH, Berlin; Germany), fluorescence optical imaging (FOI) has been shown to detect inflammation in preclinical studies as well as in humans in the joint regions of both hands [16–21]. The basis of the Xiralite method is the illustration of impaired microcirculation caused by the inflammatory process of arthritis. For that, the enhancement of the intravenously applied dye indocyanine green (ICG) is evaluated. Accordingly, FOI is a non-ionising technique that can examine both hands in a single session of 6 min.

The aim of this study was to investigate FOI’s ability to monitor the treatment response to TNFα inhibitor therapy with certolizumab pegol (CZP) in RA patients and to compare FOI findings to clinical outcome parameters such as DAS28 (CRP), tender joint count (TJC-28), swollen joint count (SJC-28) and laboratory parameters. The US7 score was set as a further imaging method with high sensitivity to inflammation.

## Patients and methods

Patients with RA according to 2010 ACR/EULAR classification criteria [22] have been enrolled after written informed consent to participate in the study. They were examined at baseline (before CZP) and after 6, 12, 24 and 52 weeks. Eligible for participation in the study were adults (age of ≥ 18 years), CZP-naïve (not naïve for other biologic therapies) patients with active RA (DAS28 (CRP)) with tolerability of the drug’s ingredients and no known heart failure (NYHA III/IV).

### Clinical and laboratory examination

During all visits, the disease activity score 28 (DAS28) [23, 24] including clinical assessments of tender (TJC-28), swollen joints (SJC-28) and patient’s self-reported global disease activity on a visual analogue scale (VAS) from 0 to 10 cm was assessed. Furthermore, physician’s global assessment (PGA) and morning stiffness (in minutes) were evaluated. The laboratory investigation included the assessment of erythrocyte sedimentation rate (ESR; normal value < 20 mm/h) and C-reactive protein (CRP; normal value < 5.0 mg/l). Clinical and laboratory examination was accomplished on the same day as the imaging examinations (FOI; US7 score).

### Fluorescence optical imaging (FOI)

The FOI system (Xiralite) was used according to a standardised procedure with an examination term of 6 min, recording one image per second, and resulting in a cluster of 360 images. One single bolus of indocyanine green (ICG) as fluorescence optical dye with a dose of 0.1 mg/kg body weight was injected intravenously ten seconds after beginning of the examination [20, 21]. To evaluate the distribution of ICG, the image sequence in the film modus was evaluated on one hand. For this purpose, three phases in position to the fingertips were defined regarding development of signal intensities that depend on the dye flooding of the individual patient [21]. Phase 1 (P1) includes the period between starting the examination, application of the dye and increased signal intensities in the fingertips [21]. When the dye leaves the fingertips from distal to proximal, phase 2 (P2) begins as the period of persisting high signal intensities in the fingertips [21]. It can be identified by the red colour in the fingertips. Phase 3 (P3) starts when no signal intensity can be determined in the fingertips, i.e. the time point without signal intensity (only yellow sparkles) in the fingertips as signal for clearance [21]. In addition, an electronically generated composite image (Prima Vista Mode, PVM) automatically obtained by means of 240 images with the integrated software XiraView was analysed.

For analysing the joint activity shown by FOI, ‘FOIAS’ (fluorescence optical imaging activity score) as a semiquantitative grading system was applied, based on individual joint scores from 0 to 3. The scores in P1-3 and in PrimaVistaMode (PVM) were assessed for 30 joints per patient, including the bilateral wrist, metacarpophalangeal joints (MCP) I-V, proximal interphalangeal joints (PIP) II-V, distal interphalangeal joints (DIP) II-V and interphalangeal joint of the thumb (IP). In addition, the sum scores of all affected joints of the left hand and the right hand were individually calculated and represented as one sum score (sum score both hands) [25]. FOIAS was performed by an agreement-based consensus of two investigators (SO, SH).

### Musculoskeletal ultrasound (MSUS)

Ultrasonographic examination was performed in greyscale (GS) and power Doppler (PD) mode with a linear 10-18 MHz transducer (Esaote Mylab Twice,Genova; Italy). The examined level of the wrist included the dorsomedian, palmomedian and ulnar plane. The metacarpophalangeal (MCP) 2,3, the proximal interphalangeal (PIP) 2,3 and the metatarsophalangeal (MTP) 2,5 joints were examined from the palmar/plantar and dorsal view in the longitudinal plane. Tenosynovitis was measured on longitudinal and transverse scans only at the wrist and MCP 2,3 joint level. The wrist, MCP/PIP2,3 and MTP 2,5 joints were examined for synovial effusion and/or proliferation (indicating synovitis) according to Scheel et al. [26] in GSUS and according to Szkudlarek et al. [27] in PDUS (each 0–3) and for signs of tenosynovitis in GS (0–1)/PDUS (0–3) [15]. The US7 score was used in a modified way also including MCP2,3 and PIP2,3 from the dorsal view in GS [28]. The scoring range was 0–39 for GS and PD synovitis, 0–5 for GS tenosynovitis (0-5) and 0–15 for PD tenosynovitis

### Statistical analyses

A two-sided Wilcoxon signed rank test was used to explore the difference in central tendency of the clinical data, FOI and MSUS scores (each 0–3, except tenosynovitis in GS US7 score) between baseline and follow-up visits. In addition, we examined whether the FOI was correlated with the clinical outcome using Spearman correlation, since a considerable number of tied values were to be expected. Furthermore, the resulting correlation coefficients were tested for their difference from zero, i.e. no correlation. Statistical analyses were performed with the statistical program SPSS. If not specified otherwise, the descriptive statistics provided median values (min–max; interquartile range [IQR]). For FOI scores, the sum of all joints and both hands were evaluated (sumscore both hands). For US7 scores, the sum of all subscores were evaluated. Whenever meaningful, *p*-values were provided based on the underlying methods. These explorative *p*-values were used to highlight statistical noticeable differences.

## Results

In this study, 28 patients with RA were included and treated with 400 mg CZP s.c. at weeks 0, 2 and 4 and every 2 weeks thereafter with 200 mg s.c.. Of these patients, all received CZP for at least 12 weeks, *n* = 27 (96.4%) until w24, and *n* = 18 (64.3 %) received CZP throughout the entire 52 weeks. The reasons for drop-out were withdrawal of CZP due to lack of efficacy (*n* = 9) or worsening of symptoms (*n* = 1). Fifteen patients were biologic naïve at inclusion. Thirteen patients had a history of biologic treatment. Sixteen patients were on background therapy with methotrexate, who received a mean dosage of 16 ± 4.28 mg per week and 23 patients with prednisolone. During the course of the study, the mean dosage of prednisolone decreased from 6.0 ± 4.75 mg per day to 3.9 ± 2.60 mg/d at w24 and 2.3 ± 2.26 mg/d at w52.

Patients’ characteristics at baseline are presented in Table 1.

**Table 1:** Baseline characteristics

	***N***	
Age (years)	28	52.5 (22–75; 20.0)
Sex (female), *n* (%)	28	26 (92.9)
Weight (kg)	28	75.5 (50–105;24.3)
Disease duration (months)	28	31.5 (4–180)
***Medication***		
Methotrexate, *n* (%)	28	16 (57.1)
Prednisolone, *n* (%)	28	23 (82.1)
Biologics naïve, *n* (%)	25^a^	15 (60.0)
***Clinical parameters***		
DAS28 (CRP)	28	4.61 (2.65–6.49; 1.75)
Tender joint count (28-TJC)	28	7.0 (0–22;8.75)
Swollen joint count (28-SJC)	28	2.5 (0–9;4.0)
VAS patient (0–10 cm)	28	6.0 (2.4–10;3.0)
VAS physician (0–10 cm)	28	4.0 (1.9–8.5;1.7)
Morning stiffness (min.)	28	30 (0–240;63.75)
***Laboratory parameters***		
ESR (mm/h)	28	27 (8–66;21.0)
CRP (mg/l)	27^b^	5.4 (0.3–52;17.4)
***Fluorescence optical imaging***^**c**^		
P1	28	1.5 (0–16;9.25)
P2	28	19.5 (0–36;14.5)
P3	28	1.0 (0–20;3.75)
PVM	28	5 (0–27;6.25)
***Musculoskeletal ultrasound (US7 Score)***		
GS US7 synovitis (range 0–39)	28	11 (4–22;8.25)
PD US7 synovitis (range 0–39)	28	2.0 (0–17;3.0)
GS US7 tenosynovitis (range 0–5)	28	0.5 (0–3;1.0)
PD US7 tenosynovitis (range 0–15)	28	0 (0–2;1.0)

Data represent median (min–max; interquartile range) unless otherwise stated. ^a^Status for 3 patients unknown; ^b^one sample not analysed; ^c^sum scores of both hands. *DAS28* disease activity score of 28 joints, *CRP* C-reactive protein, *VAS* visual analogue scale, *ESR* erythrocyte sedimentation rate, *GS* grey scale, *PD* power Doppler, *P* phase, *PVM* prima vista mode, *US7* ultrasound score of 7 selected joints

### Clinical and laboratory outcome parameters

Disease activity measured by DAS28 (CRP) continuously decreased from baseline to w52. At baseline, patients had a moderate to high disease activity with a median (min-max; IQR) DAS28 (CRP) of 4.6 (2.7–6.5; 1.8), which decreased under treatment to 3.2 (1.3–6.4;1.8; *p* = 0.001) at w24 and to 2.7 (1.2–4.9; 2.1; *p* < 0.001) at w52.

Similar results were observed for SJC-28 and TJC-28, which are also part of the DAS28 (CRP). Both outcome parameters continuously decreased from baseline to w52. For SJC-28, a decrease from median of 2.5 (0–9, 4.0) at baseline to 0.0 (0–6, 2.0; *p* = 0.005) at w24 and to 0.0 (0-6–1.8; *p* = 0.012) at w52 was observed. For TJC-28, a decrease from median of 7.0 (0–22, 8.8) at baseline to 3.0 (0–17, 4.0; *p* = 0.001) at w24 and to 2.0 (0–15, 3.5; *p* = 0.004) at w52 was observed.

Patient global assessment (VAS) decreased during the course of the study. Significant differences were observed at w6, w12 and w52. Similar results were observed for the global assessment performed by the physician.

Morning stiffness decreases continuously from baseline to w52 with significant findings at w6, w12 and w24.

CRP and ESR values continuously decreased from baseline to w52. The median CRP decreased from 5.4 mg/l (0.3–52; 17.4) to 2.3 mg/l (0.3–41; 5.5; *p* = 0.028) at w24 and to 2.0 mg/l (0.2–99; 5.0; *p* = 0.156) at w52. The median ESR decreased from 27 mm/h (8–66; 21) to 21 mm/h (2–70; 25.5; *p* = 0.203) at w24 and to 18 mm/h (2–54; 20; *p* = 0.177) at w52.

A summary of all measured clinical and laboratory outcome parameters is listed in Table 2.

**Table 2:** Summary statistics of clinical and laboratory outcome parameters at baseline and after w6, w12, w24 and w52

Clinical outcome parameters	***N***	Median (min–max, IQR)	***p***-value
***DAS28 (CRP)***			
Baseline	28	4.61 (2.65–6.49, 1.76)	
w6	28	3.80 (1.49–6.60, 1.88)	0.002
w12	27	3.26 (1.39–6.65, 2.04)	< 0.001
w24	27	3.20 (1.27–6.37, 1.81)	0.001
w52	18	2.73 (1.22–4.85, 2.06)	< 0.001
***Tender Joint Count-28***			
Baseline	28	7.00 (0–22, 8.75)	
w6	28	3.00 (0–22, 7.00)	0.009
w12	28	4.00 (0–21, 5.00)	< 0.001
w24	27	3.00 (0–17, 4.00)	0.001
w52	18	2.00 (0–15, 3.50)	0.004
***Swollen Joint Count-28***			
Baseline	28	2.50 (0–9, 4.00)	
w6	28	2.00 (0–6, 2.00)	0.052
w12	28	1.00 ((0–7, 3.00)	0.009
w24	27	0.00 (0–6, 2.00)	0.005
w52	18	0.00 (0–6, 1.75)	0.012
***CRP (mg/l***)			
Baseline	28	5.40 (0.30–52.00, 17.36)	
w6	27	4.00 (0.30–61.87, 11.85)	0.687
w12	26	3.25 (0.30–28.90, 6.95)	0.091
w24	25	2.30 (0.30–41.40, 5.50)	0.028
w52	18	2.00 (0.20–98.70, 5.02)	0.156
***ESR (mm/h)***			
Baseline	28	27 (8–66, 21,00)	
w6	28	20 (4–52, 24,50	0.027
w12	28	18 (6–56, 23.25)	0.059
w24	27	21 (2–70, 25.50)	0.203
w52	18	18 (2–54, 20.00)	0.177
***VAS (0–10 cm) patient***			
Baseline	28	6.00 (2.40–10.00, 3.00)	
w6	28	2.90 (0.00–8.50, 3.95)	0.003
w12	28	4.20 (0.10–8.90, 3.00)	0.003
w24	27	5.00 (0.30–9.00, 3.70)	0.112
w52	18	2.95 (0–8.00, 3.63)	0.002
***VAS (0–10 cm) physician***			
Baseline	28	4.00 (1.9–8.50, 1.70)	
w6	28	3.00 (1.0–7.50, 2.00)	0.002
w12	28	2.25 (1.0–8.00, 1.63)	0.001
w24	27	2.00 (0.5–10.00, 2.25)	0.066
w52	18	1.50 (0.0–6.00, 2.00)	0.014
***Morning stiffness (min.)***			
Baseline	28	30.00 (0–240, 63,75)	
w6	28	2.50 (0–120, 30.00))	0.003
w12	28	7.50 (0–90, 33.75)	0.025
w24	27	0.00 (0–120, 45.00)	0.042
w52	18	0.00 (0–150, 12.50)	0.091

*IQR* interquartile range, *DAS28* disease activity score of 28 joints, *ESR* erythrocyte sedimentation rate, *CRP* C-reactive protein, *VAS* visual analogue scale; significance level of *p* < 0.05

### Fluorescence optical imaging (FOI)

The sum score of both hands in P1 of FOI continuously decreased from baseline to w52. A decrease in P1 of FOI sum score was already observed at w6, with a reduction from a median of 1.5 (0–16, 9.3) at baseline to 1.0 (0–12, 3; *p* = 0.069) at w6. The FOI P1 1 sum score further decreased to 0.5 (0–46, 3; *p* = 0.171), 0.0 (0-–0, 3; *p* = 0.004) and 0.0 (0–13, 2.8; *p* = 0.091) at w12, w24 and w52, respectively.

A statistically significant reduction was detected at w24. Although the sumscore from baseline to w52 decreased, the applied test did not yield a significant result. This likely occurred due to the reduced number of patients at that time point.

All other sum scores of both hands (PVM, P2 and P3) did not show a median reduction during the treatment.

The results of the FOI are presented in Table 3.

**Table 3:** Summary statistics of FOI phases 1-3 and PMV at baseline, w6, w12, w24 and w52

	***N***	Median (min-max, IQR)	***p***-value
***Phase 1***			
Baseline	28	1.5 (0–16, 9.25)	
w6	27	1.0 (0–12, 3.00)	0.069
w12	28	0.5 (0–46, 3.00)	0.171
w24	27	0.0 (0–10, 3.00)	0.004
w52	18	0.0 (0–13, 1.75)	0.091
***Phase 2***			
Baseline	28	19.5 (0–36, 14.50)	
w6	27	19.0 (0–43, 13.50)	0.451
w12	28	19.0 (0–50, 13.25)	0.071
w24	27	15.0 (1–48, 15.50)	0.310
w52	18	19.0 (0–35, 12–75)	0.256
***Phase 3***			
Baseline	28	1.0 (0–20, 3.75)	
w6	27	2.0 (0.22, 3.50)	0.501
w12	28	2.5 (0–32, 6.25)	0.089
w24	27	1.0 (0–17, 3.00)	0.191
w52	18	0.5 (0–12, 2.00)	0.959
***PMV***			
Baseline	28	5.0 (0–27, 7.00)	
w6	27	8.0 (0–25, 7.00)	0.018
w12	28	5.5 (0–36, 9.25)	0.472
w24	27	7.0 (0–23, 8.50)	0.204
w52	18	5.5 (0–23, 5.75)	0.717

*FOI* fluorescence optical imaging, *PMV* PrimaVistaMode, *IQR* interquartile range; significance level of *p* < 0.05

**Fig. 1:**
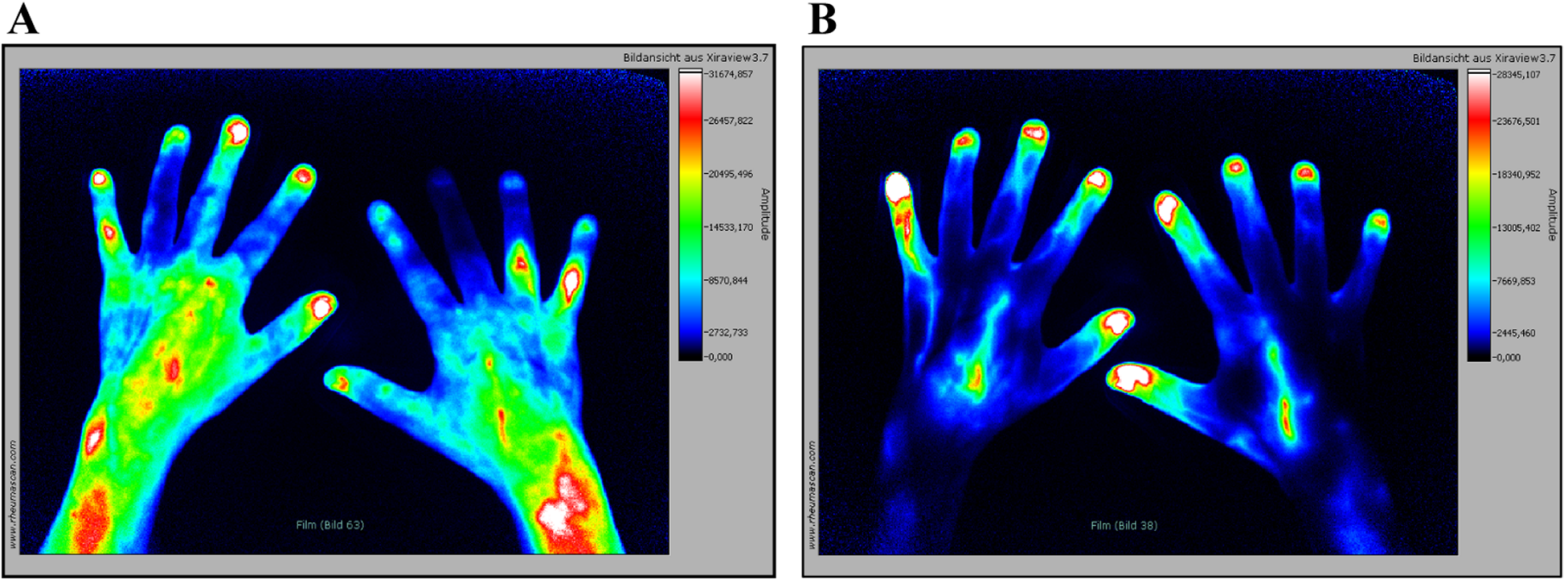
Fluorescence optical imaging illustrations in phase 1. **A** Baseline visit. FOI findings with clinical active RA. Increased signal intensities as a sign of active inflammation in P1. High signal intensities (FOIAS grade 3) in PIP5 of the right hand, moderate signal intensities (FOIAS grade 2) in both wrists and PIP4 of the right and PIP5 of the left hand. **B** After 52 weeks of CZP treatment; no increased signal intensities are detectable

Figure 1 shows an example of a patient with good response to the CZP therapy which is displayed as a signal attenuation in comparison from baseline, visit 1 to week 52 in P1. The improvement of the inflammatory process can be seen in the significant decrease of early enhancement in both hands during the treatment period.

### Musculoskeletal ultrasound

For GS, a numerical decrease of the US7 synovitis sum score was only observed at w52 (from 11.0 at baseline to 8.5 at w52).

For PD, a numerical decrease of the US7 synovitis score was detected at w12 (from 2.0 to 1.5) with a further decrease at w52 (to 0.5).

For GS tenosynovitis und PD tenosynovitis US7 scores, no relevant changes could be detected throughout the study.

The outcome parameters of the GS and PD US7 synovitis/tenosynovitis scores are presented in the Supplement Table S1 at baseline and after 6, 12, 24 and 52 weeks of therapy with CZP.

A visual summary of the results is provided in Supplement Figure S2 which presents box plots of DAS28, FOIAS P1, GS, PD, SJC-28 and TJC-28 at baseline, w6, w12, w24 and w52.

### Correlation of FOIAS with selected clinical and US7 score outcome parameters

Regarding baseline data, FOIAS in PMV and P1, P2 P3 showed no significant positive correlations with any clinical outcome parameter. There was a moderate correlation between FOIAS P3 with PD US7 synovitis score. All other US7 Score parameters showed a weak or no positive correlation with FOIAS in PMV and P1, P2, P3.

At week 24, there was a moderate correlation between FOIAS in P2 with DAS28 and with TJC-28. All other clinical parameters showed weak or no positive correlation with FOIAS in PMV and P1, P2, P3. No significant correlation could be presented between FOIAS in PMV and P1, P2, P3 with all US7 Score parameters.

At week 52, FOIAS P2 showed a moderate correlation with TJC-28. There was a moderate correlation between FOIAS P2, P3, PVM and PD synovitis score and a moderate correlation between FOIAS P3, PVM and PD tenosynovitis score.

Pairwise correlations between clinical and laboratory outcomes with FOIAS P1-P3 and PMV as well as with US7 Score parameters are presented in Figure S1 (supplement).

## Discussion

In this open-label study, we are presenting for the first time that FOI assessments by means of short-term study visits allow early monitoring of treatment response after initiation of anti-TNFα therapy. Already after a 6-week treatment period, FOI in P1 showed a reduction of ICG enhancement based on the FOIAS and at w24; the reduction of the FOIAS compared to baseline was statistically significant (*p* = 0.004). However, at w52, no significant reduction of the FOIAS compared to baseline has been observed—most probably due to the low number of patients (*n* = 18) at w52. Furthermore, comparing the results between w24 and w52, no additional effect in the FOIAS could be observed. The reason is unclear but could indicate a total remission of inflammation already seen after w24. However, the sample size is small and further studies are necessary to draw a final conclusion.

According to the results of this analysis, FOI in P1 seems to be the most useful FOI phase for monitoring treatment response.

These results are in line with previous results by Meier et al. [29] and Glimm et al. [25] presenting significant reductions in early signal intensities after 24 weeks and after 1 year of treatment, respectively. In addition, in the study of Glimm et al., FOI P1 was the only FOI phase with significant changes over the treatment period of 1 year. In another study on osteoarthritis [17] as the primary non-inflammatory joint disease, OA patients exhibited less activity in FOI P 1 compared to joint inflammation in RA (though, high signal accumulation in P2). These results support the hypothesis that FOI P1 detects an acute inflammation.

Regarding clinical disease activity, DAS28 (CRP) decreased significantly from a median of 4.6 to a median of 3.2 at w24. After 52 weeks of treatment, a low disease activity (median of 2.7) was observed. The good response of CZP treatment in RA patients could also be shown by a reduction of the prednisolone dosage, which decreased from 6.0 ± 4.75 mg per day to 2.3 ± 2.26 mg/day at w52.

A correlation between DAS28 (CRP) and P1 could not be observed, which again is in line with the findings by Glimm et al. [25]. The lack of correlation may be caused by measuring different outcome parameters assessed through the clinical examination by DAS28 compared to the FOI method. Clinical disease activity is mainly assessed by swollen and tender joints and by patients’ self-reported VAS. In contrast, FOI via the Xiralite method detects disturbed microcirculation in the joints of both hands. Hypervascularisation and neoangiogenesis of the synovial membrane are considered to be primary pathogenic mechanisms for the development of rheumatoid pannus on the joint leading to bone destruction [30].

Because of the subjective component of DAS28 (CRP) in decision making during RA treatment, which leads to a high variance of the DAS28 (CRP) score, FOI represents a more objective method for therapy monitoring.

Another explanation for the lack of correlation between FOI P1 and clinical outcome parameters could be the positive findings in FOI in asymptomatic joints, indicating subclinical inflammation. Werner et al. [20] found that FOI showed positive findings in 45% of clinically asymptomatic joints.

In our study, we also included the US7 score for detection of synovitis and tenosynovitis. The US7 synovitis sum score decreased, but not initially after start of CZP therapy. For GS, a numerical decrease of the synovitis sum score was observed only at w52 and for PD, a decrease of the synovitis score was detected at w12 with further decrease at w52. Sarzi-Puttini et al. [31] already found an improvement in PD ultrasound after w8 of CZP therapy, which continued to w52. Overall, the MSUS results are in line with other studies. In contrast to the fast response in PD scoring, which identifies active inflammation, the GS usually identifies chronic or later changes such as synovial proliferation, which explains the late response in our study [15, 32–34]. In spite of the positive results in clinical studies, the operator-dependency and time-consumption are potential limitations of MSUS [33].

An advantage of FOI is the ability to do fast imaging in a comfortable sitting position by using a machine which can be installed in every clinical setting. It is also a delegable tool that can performed by a medical assistance with a physician in the background. FOI can be used in both diagnostic and therapy monitoring setting. Therapy monitoring by means of short-term FOI study visits is likely the more important role because biological therapies such as anti-TNFα therapies are highly effective but expensive. Thus, to keep costs as low as possible and to provide the patient with the optimal treatment, it is important to obtain early objective evidence of a response or non-response.

The use of the chosen imaging method depends on the treating physician’s preference and the indication for imaging. Information on bone erosions will likely remain the domain of MRI, MSUS and X-ray. However, determination of the degree and extent of joint inflammation as well as for the diagnostic of subclinical inflammation are indication for the use of FOI method. Because of the detection of early synovitis in symptomatic and asymptomatic patients, FOI might also be a tool in early clinical studies to detect treatment response.

A limitation of the current study is the open design and the small sample size. Treatment responders could also be influenced by other factors that were not considered into the analysis, e.g. wounds located over the joint. Another limitation of the FOI device is that the system can only image the hands, so far. Furthermore, the intravenous ICG administration before initiating the FOI procedure might limit the routine use in clinical practice.

## Conclusion

To our knowledge, this is the first study showing that FOI can be used to monitor early treatment response after initiating anti-TNFα therapy. Significant reductions in FOIAS were found in P1 which appears to be the most relevant phase for monitoring treatment response.

In contrast to clinical outcome parameters, FOI represents—in our opinion—a more objective method for therapy-monitoring and may provide an additional tool to assess early therapy response after initiating therapy with biologics. It may also provide the opportunity for an additional evaluation of inflammation of wrist and finger joints of RA patients in clinical studies. Consequently, decrease in disease activity can easily be monitored with the Xiralite system by quantifying the fluorescence signals in the early phase (= phase 1) of signal acquisition.

Fluorescence optical imaging with the Xiralite system is an interesting alternative to other imaging methods, not only in terms of providing a fast and easy assessment but also its pictorial output, making it easier for patients to follow and understand, enabling higher acceptance of treatment measures.

## Data Availability

The datasets used and/or analysed during the current study are available from the corresponding author on reasonable request.

## Abbreviations

ACR: American College of Rheumatology
ACPA: Anti-cyclic citrullinated peptides
bDMARD: Biological disease-modifying anti-rheumatic drug
CRP: C-reactive protein
csDMARD: Conventional synthetic disease-modifying anti-rheumatic drug
CZP: Certolizumab pegol
DAS28: Disease activity score of 28 joints
ESR: Erythrocyte sedimentation rate
EULAR: European alliance of associations for rheumatology
FOI: Fluorescence optical imaging
FOIAS: Fluorescence optical imaging activity score
GS: Greyscale (ultrasound)
ICG: Indocyanine green
IgM-RF: Immunoglobulin M-rheumatoid factor
IP: Interphalangeal joint of the thumb
IQR: Interquartile range
MCP: Metacarpophalangeal (joint)
MRI: Magnetic resonance imaging
MSUS: Musculoskeletal ultrasound
MTP: Metatarsophalangeal (joint)
NSAIDs: Non-steroidal anti-inflammatory drugs
NYHA: New York Heart Association
OMERACT: Outcome measures in rheumatology clinical trial
P: Phase
PGA: Physician’s Global Assessment
PIP: Proximal interphalangeal (joint)
PD: Power Doppler (ultrasound)
PVM: PrimaVistaMode
RA: Rheumatoid arthritis
RMD: Rheumatic and musculoskeletal diseases
SJC-28: Swollen joint count-28
TNFα: Tumour necrosis factor α
TJC-28: Tender joint count-28
US7 Score: Ultrasound Seven Score
VAS: Visual analogue scale
